# AGEs-Induced and Endoplasmic Reticulum Stress/Inflammation-Mediated Regulation of GLUT4 Expression and Atherogenesis in Diabetes Mellitus

**DOI:** 10.3390/cells11010104

**Published:** 2021-12-29

**Authors:** Marisa Passarelli, Ubiratan Fabres Machado

**Affiliations:** 1 Laboratório de Lípides (LIM-10), Hospital das Clínicas (HCFMUSP) da Faculdade de Medicina da Universidade de São Paulo, São Paulo 01246-000, Brazil; m.passarelli@fm.usp.br; 2 Programa de Pos-Graduação em Medicina, Universidade Nove de Julho, São Paulo 01525-000, Brazil; 3 Department of Physiology and Biophysics, Institute of Biomedical Sciences, University of São Paulo, São Paulo 05508-000, Brazil

**Keywords:** diabetes mellitus, hyperglycemia, endoplasmic reticulum stress, GLUT4, atherogenesis, cardiovascular disease, advanced glycation end product

## Abstract

In recent decades, complex and exquisite pathways involved in the endoplasmic reticulum (ER) and inflammatory stress responses have been demonstrated to participate in the development and progression of numerous diseases, among them diabetes mellitus (DM). In those pathways, several players participate in both, reflecting a complicated interplay between ER and inflammatory stress. In DM, ER and inflammatory stress are involved in both the pathogenesis of the loss of glycemic control and the development of degenerative complications. Furthermore, hyperglycemia increases the generation of advanced glycation end products (AGEs), which in turn refeed ER and inflammatory stress, contributing to worsening glycemic homeostasis and to accelerating the development of DM complications. In this review, we present the current knowledge regarding AGEs-induced and ER/inflammation-mediated regulation of the expression of GLUT4 (solute carrier family 2, facilitated glucose transporter member 4), as a marker of glycemic homeostasis and of cardiovascular disease (CVD) development/progression, as a leading cause of morbidity and mortality in DM.

## 1. Introduction

Diabetes mellitus (DM) is a global public health burden, as the number of affected adults is expected to rise to 630 million by 2045 [1]. DM is a metabolic disorder characterized by inappropriate hyperglycemia and can be classified into type 1 DM (T1D), which primarily results from the lack of insulin secretion, and type 2 DM (T2D), which primarily results from insulin resistance (IR) [2]. However, whereas T2D subjects develop impaired insulin secretion, T1D subjects may also develop IR, as a complex result of interactions among age, sex, pregnancy, ethnicity, duration of disease, increased life expectancy, genetic predisposition, epigenetic changes, glucotoxicity, lipotoxicity, overweight and intensive insulin therapy [2,3,4,5]. In addition to glycemic disorder, chronic hyperglycemia injures various organic systems, leading to the development of several complications, prompting a 2- to 4-fold increase in the risk of cardiovascular disease (CVD) [6,7].

In DM, several metabolic mechanisms, notably hyperglycemia, lead to the activation of intracellular pathways that increase the generation of advanced glycation end products (AGEs); thus, increased plasma level of AGEs has become a hallmark of uncontrolled DM [8]. In turn, AGEs contribute to worsening IR and glycemic control [9], and participate in the development of chronic diabetic complications, including CVD [10]. The receptor for AGEs—AGER (advanced glycation end product-specific receptor, formerly RAGE)—mediates several biological effects of AGEs, including the activation of endoplasmic reticulum stress (ER stress) and inflammatory stress pathways [10].

Considering that AGEs can participate in both the impairment of glycemic homeostasis and the development/progression of chronic complications of DM, and considering that these effects involve ER stress- and inflammatory-mediated pathways, the present manuscript reviews these regulations upon the expression of solute carrier family 2, facilitated glucose transporter member 4 (GLUT4) expression, as a marker of altered glycemic homeostasis [11,12,13] and, upon atherogenesis, as an important inducer of the development/progression of CVD [7].

Names and symbols of genes and proteins referred to in this manuscript are in accordance with the HGNC (https://www.genenames.org/, accessed on 2 November 2021) and the UNIPROT (https://www.uniprot.org/, accessed on 2 November 2021) databases. Whenever necessary, alias symbols will be provided when they first appear in the text.

## 2. Pathogenesis of DM and Its Complications

### 2.1. Insulin Resistance (IR)

Insulin resistance involves complex and variable disturbances in the insulin signaling pathway, eventually culminating in impaired glucose utilization by muscle and adipose tissues, contributing to the impairment of glycemic control [12,14]. Furthermore, IR aggravates hyperglycemia by favoring the output of glucose from the liver by increasing gluconeogenesis. Compensatory hyperglycemia, in turn, is not able to neutralize hepatic glucose production, whereas, in a non-resistant pathway, induces an increase in lipogenesis mediated by the overactivation of sterol regulatory element-binding protein 1 (SREBP1), which aggravates peripheral insulin resistance and favors a pro-atherogenic state [15]. Then, plasma glucose levels are dictated by an interplay of mechanisms triggered by IR and hyperinsulinemia in one or more different organs.

Glucose uptake in muscle and adipose tissues depends on the insulin-responsive glucose transporter GLUT4, codified by the *SLC2A4* gene (solute carrier family 2 member 4), which was cloned 30 years ago [16]. Since then, it has been clear that GLUT4 plays a fundamental role in plasma glucose clearance and glycemia homeostasis as well [11,12]. Furthermore, alterations in GLUT4 expression have been related to alterations in glycemic control, with reduced GLUT4 content participating in the hyperglycemia prone. Because of that, in the present manuscript, muscle and adipose tissues regulation of *SLC2A4*/GLUT4 is reviewed as a marker of glycemic control in DM.

On the other hand, combinations of IR and compensatory hyperinsulinemia (in T2D) or with hyperinsulinemia induced by insulin therapy (in T1D) have also been reported as capable of causing damage to various organ systems. This leads to the development of disabling and life-threatening health complications, most of which are microvascular (retinopathy, nephropathy and neuropathy) and macrovascular complications (CVD, cerebrovascular disease and peripheral artery disease) [7].

Insulin resistance and hyperinsulinemia have been associated with polycystic ovary syndrome, nonalcoholic fatty liver disease, certain forms of cancer, sleep apnea and, especially, with hypertension and CVD [17,18]. Related to that, several disorders that course with IR have been considered as risk factors for the development of hypertension and CVD, including a high atherogenic lipoprotein profile (in its complexity), elevated plasma concentrations of plasminogen activator inhibitor-1, increased sympathetic nervous system activity and endothelial disfunction [17,18]. Along with these risk factors, a pro-inflammatory profile and the activation of reticulum endoplasmic stress and oxidative stress are observed, in a complex relationship, creating a dangerous vicious circle [19,20]. Because of that, in this manuscript, CVD (with special focus on atherogenesis) was chosen to be reviewed as the most important complication of DM.

### 2.2. GLUT4 Expression and Glycemic Control

The glucose transporter protein GLUT4 belongs to a family of proteins responsible for the glucose facilitative diffusion across the plasma membrane (PM) (for a review, see [21]). GLUT4 is the only insulin-sensitive glucose transporter, which is mainly expressed in the skeletal muscle and adipose tissue, where it is responsible for the insulin-induced glucose uptake. In myocytes and adipocytes, the binding of insulin in its receptor triggers the activation of an exquisite intracellular sorting of signals that, eventually, culminates in a GLUT4 storage vesicle translocation to the PM. After docking and fusing events, the amount of GLUT4 in PM increases, enhancing the glucose influx. Since intracellular consumption of glucose is high in these cells, the diffusion gradient continuously favors the influx of the substrate. Disruption of the insulin stimulus leads to the internalization of GLUT4, restoring the glucose uptake to basal levels (for a review, see [16]). The GLUT4-mediated increase in glucose uptake in muscle and adipose tissues is a fundamental mechanism involved in the blood glucose clearance, especially in the postprandial state. Thus, several research groups have investigated the regulation of *SLC2A4* gene expression, which codifies the GLUT4 protein, for better understanding the insulin-mediated plasma glucose clearance [13,22,23].

Impairment of insulin signaling transduction can compromise GLUT4 translocation to the PM, notably in acute situations, in which the total cellular GLUT4 content is unchanged. However, under chronic insulin-resistant states, a decreased total GLUT4 content is currently observed, and that certainly contributes to decreased GLUT4 at the PM in response to insulin. Even considering a preserved translocation system, when the intracellular GLUT4 content is reduced, the final amount of GLUT4 at the PM will be reduced [24]. This emphasizes the great relevance of the repression of *Slc2a4* gene expression, and consequent reduction in GLUT4 protein, in the IR and DM states. Besides, the role of *Slc2a4*/GLUT4 expression in glycemic control has been reinforced by studies with transgenic mice. It is well recognized that *Slc2a4* knockout induces hyperglycemia, whereas the overexpression of *Slc2a4* improves glycemic control even in DM mice [25,26], regulations that were directly associated with the amount of GLUT4 at the PM, independently of changes in insulin signaling. These data reinforce the importance of the regulation of *SLC2A4* gene expression in glycemic control and qualify this gene as a promising target for the pharmacogenomics of IR/DM [12].

At present, several transcriptional factors are described as involved in the expression of *SLC2A4*/*Slc2a4* (human/murine genes, respectively), most of them acting as enhancers and a few as repressors (for a review, see [22,23]). Importantly, some of these transcription factors have been related to the inflammatory and/or ER stress activity, characterizing these routes as important modulators of GLUT4 expression, as will be commented on next.

### 2.3. Cellular Cholesterol Homeostasis and Cardiovascular Disease (CVD) Development

Cardiovascular disease is the most prevalent cause of disability and morbimortality in DM [27]. Although CVD is prevalent in both T1D and T2D, it is especially of greater concern in T2D, which comprises about 91% of all DM cases. Insulin resistance and compensatory hyperinsulinemia, obesity, chronic inflammation and derangements in lipid metabolism that contribute to lipid accumulation in the arterial wall favor the clinical burden of CVD complications. Moreover, the presence of other CVD risk factors commonly associated with metabolic syndrome increases atherogenesis [28].

In T2D, quantitative changes of plasma lipoproteins occur as a primary consequence of the insulin-resistant state [29]. Hyperinsulinemia increases hepatic triglycerides synthesis by overactivating the SREBP1. This transcriptional factor resides in the ER membrane and its transference to the Golgi compartment is controlled by hormonal and nutritional signaling. Then, the small fragment containing the leucine zipper, released after two consecutive proteolytic cleavages, enters the nucleus, where it induces the transactivation of genes involved in the de novo synthesis of fatty acids. Moreover, increased lipolysis favors the free fatty acids flow from the adipose tissue to the liver, allowing more substrate for triglycerides synthesis. These events contribute to a greater secretion of very-low-density lipoprotein (VLDL), which in the blood is less metabolized by the lipoprotein lipase, contributing to hypertriglyceridemia. The impairment of triglyceride-rich lipoprotein lipolysis reduces the generation of high-density lipoprotein (HDL). Furthermore, small and dense low-density lipoprotein (LDL), generated according to the increase in plasma triglycerides levels, characterizes a more atherogenic particle that has more access to the arterial wall, being susceptible to oxidation. Monocyte-derived macrophages take up oxidized LDL, contributing to the first stage of atherosclerotic plaque formation. The increased generation of oxysterols accompanies the intracellular accumulation of cholesterol, eliciting inflammation [30].

The balance between cholesterol supply to arterial wall cells (mainly via modified LDL) and lipid removal (by HDL) dictates the amount of accumulated lipids that modulates atherogenesis. Reverse cholesterol transport is the centripetal flux of cholesterol that allows its excretion into bile and feces, characterizing the major driving force of cholesterol out of the arterial wall compartment. Lipid-free apolipoprotein A-1 (APOA1) interacts with the phospholipid-transporting ATPase ABCA1 (ABCA1), a putative receptor for APOA1 and nascent HDL (pre-beta HDL), which drives excess cholesterol outside cells by obtaining energy from the hydrolysis of two ATP molecules. After esterification by the phosphatidylcholine-sterol acyltransferase (alias lecithin cholesterol acyltransferase), esterified cholesterol becomes part of the growing hydrophobic core of HDL, namely HDL_3_, and subsequently HDL_2_. Larger HDL interacts with the ATP-binding cassette transporter G-1 (ABCG1), which exports excess cholesterol and some oxysterols. Then, HDL can discharge esterified cholesterol directly into the liver by interacting with the scavenger receptor class B member 1 or indirectly by transferring esterified cholesterol to LDL, VLDL or chylomicrons by the action of the cholesteryl ester transfer protein. In the latter pathway, cholesterol removal is mediated by the uptake of APOB-containing lipoproteins by hepatocytes [29].

### 2.4. Advanced Glycation End Products (AGEs)

Hyperglycemia induces intracellular oxidative stress that is considered a link for all DM-related complications. Increased cellular glucose influx favors its metabolism through glycolytic pathway enhancing the electron flow in the mitochondrial respiratory chain, which relates to the generation of reactive oxygen species (ROS), particularly superoxide anion. The consequent increased expression and activity of poly (ADP-ribose) polymerase, as an adaptive mechanism that protects against DNA damage, leads to the modification of the structure of glyceraldehyde-3-phosphate dehydrogenase by poly-ADP-ribosylation. The impairment of glycolysis flow deviates glyceraldehyde 3-phosphate and dihydroxyacetone phosphate to the formation of methylglyoxal (MGO), a very reactive oxoaldehyde that interacts with proteins, phospholipids and nucleic acids, leading to the irreversible formation of AGEs [31]. Usually, under normoglycemic conditions, MGO formation occurs in very low rates, with around 0.05–0.1% of the triosephosphates degrading to MGO. In addition, its cellular concentration is low due to the action of the glyoxalase system consisting of glyoxalases 1 and 2, which convert MGO into, respectively, S-D-lactoylgluthatione and D-lactate (for a review, see [32]). In the setting of hyperglycemia, AGEs are further increased in some cells by the coactivation of the polyol pathway. Aldose reductase catalyzes glucose conversion into sorbitol, which is converted into fructose by the sorbitol dehydrogenase activity. Fructose is very reactive with proteins leading to the rapid formation of AGEs; in addition, by depleting NADPH, the polyol pathway impairs the glutathione resynthesis, favoring oxidative stress that increases the generation of AGEs [31].

In the blood circulation, excess glucose nonenzymatically interacts with the amino terminal portion of lysine and arginine residues in proteins, or with amino residues of phospholipids, leading to the formation of an unstable Schiff base. According to the maintenance of hyperglycemia, an Amadori product is generated, and after inter- and intramolecular rearrangements, AGEs are formed, including very heterogeneous compounds. Oxoaldehydes, including MGO, glyoxal, 3-deoxyglucosone and glycolaldehyde, are intermediates of this process; dicarbonyl sugars and other oxoaldehydes are more reactive than glucose and increase not only in DM, but also in chronic kidney disease, inflammation, and disorders associated with oxidative stress, leading to the rapid generation of AGEs. Moreover, oxoaldehydes increase in the postprandial period favoring a rapid modification of circulating proteins. As a consequence, there is a change in the intracellular and extracellular protein structure and functionality, affecting the extracellular matrix, cellular interactions, receptor-mediated cell responses and DNA structure. Some AGEs are intense fluorophores, allowing their measurement by fluorimetry and may induce covalent crosslinks [33]. Carboxymethyllysine (CML), carboxyethyllysine, pentosidine, pyrraline, MGO dimers, glyoxal dimers, MGO-derived hydroimidazolones and glucosepane are major species of AGEs that have been associated with long-term complications of DM [34]. AGEs, together with other metabolic pathways altered during hyperglycemia, including the polyol pathway, activation of protein kinase C and hexosamine pathways, constitute the molecular basis for cellular damage in DM (for a review, see [28]).

Advanced glycation end products interact with the receptor AGER, a multiligand receptor that activates NADPH oxidase, inducing ROS generation. This leads to the NFKB nuclear factor kappa-B (NFKB) activation and transactivation of several genes, including *AGER*, which contributes to a deleterious vicious circle. In addition, AGER overlaps with toll-like receptor signaling and can bind to other ligands, including calgranulins, serum amyloid and high-mobility group protein 1 (alias amphoterin), increasing inflammatory stress. The localization of AGER in the cell surface as well as in intracellular organelles enables signaling triggered by different ligands and both intra- and extracellularly formed AGEs [35]. Furthermore, soluble isoforms of AGER, lacking the intracellular domain of the native receptor, can bind AGEs without triggering intracellular signaling; thus, the amount of soluble AGER in blood might be a protective biomarker of DM complications risk [36].

On the other hand, the AGE receptor DDOST (dolichyl-diphosphooligosaccharide-protein glycosyltransferase, alias AGER1) antagonizes AGER signaling by inducing antioxidant genes attenuating the effects of AGEs. In T1D, a lower expression of *DDOST* in peripheral blood mononuclear cells was found in comparison to healthy controls after adjustment for sex, age, use of statins, angiotensin-converting enzyme inhibitors and angiotensin receptor blockers [37]. In this sense, the balance between AGER and DDOST expression may be detrimental when analyzing the contribution of AGEs to tissue damage in DM.

Advanced glycation impairs lipid metabolism, contributing to qualitative and quantitative alterations in plasma lipoproteins. AGEs modify both phospholipids and apolipoproteins in the lipoprotein structure, disturbing its recognition by cellular receptors and its metabolism by enzymes and proteins in the plasmatic and lymphatic compartments. VLDL and chylomicron glycation damages the activity of lipoprotein lipase; glycation of APOB increases its half-life, allowing the entrance of LDL in the arterial wall. Glycated LDL is also more oxidized and immunogenic, and for this reason, is more rapidly captured by arterial macrophages [38]. In DM, HDL modified by MGO is increased and severely compromises HDL generation, half-life and functionality, affecting cholesterol flow along the reverse cholesterol transport as well as other antiatherogenic properties of HDL [39].

## 3. Endoplasmic Reticulum Stress (ER Stress) and Inflammation in DM

Thirty years ago, the study of ER stress was initiated by investigating the cell reaction to heat shock, including the rapid induction of heat shock proteins (HSPs), such as the heat shock 70 kDa protein (HSP70) [40]. After the initial description of the HSP participation in cellular homeostasis and immune function, an exquisite ER stress pathway was rapidly characterized and pleiotropic roles have been proposed, including participation in the development of DM (for a review, see [41]).

In DM, ER stress was first associated to a protective effect of pancreatic beta cells. Despite the normal development of endocrine pancreas in eukaryotic translation initiation factor 2-alpha kinase 3 (EIF2AK3, alias PERK)-deficient mice, postnatal activation of ER stress is accompanied by increased cell death and leads to a progressive development of DM [42]. Later, it was demonstrated that inositol-requiring enzyme 1 (IRE1)-deficient mice exhibited mild hypoinsulinemia with hyperglycemia, especially after a glucose challenge, and despite an unaltered histological analysis of the pancreatic islets, a reduction in pancreas mass was described [43]. Furthermore, during the development of DM in Akita mice, overexpression of the ER-stress-related proteins BIP (endoplasmic reticulum chaperone BIP; alias GRP78) and DDIT3 (DNA damage-inducible transcript 3 protein; alias CHOP10/GADD153) was observed, and targeted disruption of the *Ddit3* gene delayed the onset of DM, confirming the participation of ER stress in beta cell damage [44].

Additionally, ER stress has also been related to the pathogenesis of IR and T2D. The development of high-fat diet-induced obesity and T2D decreases in *Hspa5* (heat shock protein family A member 5)-null mice (knockout for BIP) [45]. Repression of many UPR (unfolded protein response)-related genes also has been observed in beta cell exposed to a high concentration of glucose [46]. Indeed, multiple studies have demonstrated the involvement of UPR in the development and progression of metabolic diseases (for a review, see [47]). Thus, nowadays, it is known that ER stress participates in the impairment of insulin secretion and action; furthermore, ER stress has also been related to the development of degenerative complications (for a review, see [48,49,50]).

It was in the early 2000s that metabolic disturbances were proposed to be associated to increased inflammatory activity, due to evolutionary reasons (for a review, see [51,52]). It was demonstrated that the development of obesity was accompanied by increased production of inflammatory cytokines by the adipose tissue, defining obesity as a disease of a subclinical inflammatory activity [53]. Rapidly, it was verified that pro-inflammatory activity would be spread to other territories, participating in the pathogenesis of T2D [50,51,52].

As the molecular mechanisms triggered by inflammation were being characterized, it was becoming clear that many of them were shared with the UPR pathway, revealing an interplay between these two processes. For instance, the activation of some UPR components, such as BIP, IRE1 and TRAF2 (TNF receptor-associated factor 2), leads to IKKB/A (inhibitor of nuclear factor kappa-B kinase subunits beta/alpha) phosphorylation, triggering the activation of NFKB, an important mediator of the inflammatory activity [54]. Nowadays, it is clear that UPR is a central proteostatic pathway that can modulate both immunity and inflammation (for a review, see [55,56]).

### 3.1. GLUT4 Expression and ER Stress/Inflammation

After the suggestion that the activation of ER stress plays an important role in the pathophysiology of T2D, ER stress began to be investigated in the regulation of GLUT4 expression in adipocytes. Later, it was reported that ER stress response decreased *Slc2a4* expression at the gene transcription level, along with increased expression of the *Ddit3* gene. It is worth recalling that the DDIT3 protein is an inhibitor of the activity and expression of the *Cebpa* gene (*CCAAT enhancer binding protein alpha*), and the CEBPA transcription factor is a potent activator of *Slc2a4*/GLUT4 expression [57,58].

Studies in the muscle started in insulin-resistant mouse C2C12 myotubes, in which a mixture of black soybean peptides increased the glucose transport and GLUT4 translocation, in parallel to the inhibition of the ER stress response [59]. Further, in rat myotubes, induction of IR by glucosamine treatment was reported to activate some ER stress markers, such as BIP and XBP1(X-box-binding protein 1), and to increase the expression of the *activating transcription factor 6* (*Atf6*) gene [60]. ATF6 overproduction inhibits the expression of some important enhancers of *Slc2a4* expression, such as MEF2A (myocyte enhancer factor 2A) and PRGC1A (peroxisome proliferator-activated receptor gamma coactivator 1-alpha, alias PGC1A), thus explaining the repression of *Slc2a4*/GLUT4. Finally, it was confirmed that *Atf6* silencing (with small interfering RNA) was sufficient to completely avoid these glucosamine-induced effects. These data elegantly demonstrate that ER stress causes IR in myotubes by impairing GLUT4 expression via an ATF6-mediated pathway [60].

After the first indication that inflammation could inhibit the expression of the GLUT4 [53], we started investigations focusing on demonstrating this regulation. First, it was suggested (in soleus muscle) that in vivo metabolic conditions (such as fasting) and in vitro treatments (such as muscle contraction and incubation with insulin) alter *Slc2a4* mRNA expression, conversely to the *Nfkb* mRNA expression [61]. Electrophoretic mobility shift assay (EMSA) also revealed that the NFKB-binding activity to a consensus NFKB-binding site changes in parallel to the *Nfkb* mRNA expression [61]. These data strongly suggest that the inflammatory-induced repression of *Slc2a4* gene expression involved a NFKB-mediated transcriptional effect.

Participation of the NFKB-mediated inflammatory activity in *Slc2a4* mRNA expression was further confirmed by (1) the anti-inflammatory effect of atorvastatin in adipose tissue of T2D mice [62]; (2) the insulin sensitizer effect of inhibition of cannabinoid receptor 1 in 3T3-L1 adipocyte [63]; (3) the dose-dependent insulin-induced enhancement of the *Slc2a4* expression in soleus muscle [64]; and (4) the oleic and linoleic fatty acids-induced repression of the *Slc2a4* expression in L6 muscle cell [65]. All these studies revealed a converse regulation between the *Slc2a4* expression and the NFKB expression/activity, indicating that the NFKB transcription factor has a repressor effect upon the *Slc2a4* gene; that is very unusual considering that NFKB is currently known as an enhancer of many genes.

Importantly, the promoter region of the *Slc2a4* gene does not exhibit the consensus kappa-B-binding site; thus, we investigated a homologous sequence located at the -134/-113 region of the mouse *Slc2a4* gene. We confirmed that both the p50 and p65 subunits of NFKB bind into this region in vitro (EMSA) and in vivo (chromatin immunoprecipitation assay); moreover, a reporter gene assay confirmed that this region is responsible for inhibiting the *Slc2a4* transcription [62]. In fact, in most of our studies commented on above concerning inflammatory-induced regulation of the *Slc2a4* expression, we have confirmed the binding activity of both p50 and p65 NFKB subunits into this -143/-113 *Slc2a4* promoter region [62,63,64,65,66].

Finally, we have demonstrated the involvement of inflammation and ER stress in the repression of *Slc2a4*/GLUT4 expression in muscle cells exposed to palmitate [67]. Acute treatment increased the BIP, EIF2A (eukaryotic translation initiation factor 2A), EIF2AK3, IRE1 and TRAF2 protein content, and EIF2AK3 phosphorylation, but did not elicit EIF2A and IKK phosphorylation or increased XBP1 nuclear content; additionally, acute and chronic treatments increased the NFKB p65 nuclear content and the NFKB-binding activity in the *Slc2a4* gene promoter. All these data reveal that palmitate treatment induces the activation of the initial components of UPR, such as the formation of a IRE1–TRAF2–IKK complex, converging to a NFKB-mediated repression of *Slc2a4*/GLUT4, and revealing a link between ER stress and inflammation in IR [67].

Endoplasmic reticulum and inflammatory stress (with reduced GLUT4 expression) have been observed in insulin-resistant skeletal muscle from women with gestational DM, and suppression of ER stress by tauroursodeoxycholic acid (TUDCA) or siRNA knockdown of IRE1A and BIP protein significantly downregulated the activation of ER and inflammatory stress, and increased the GLUT4 expression and glucose uptake [68]. Interestingly, it was demonstrated that prolonged preoperative fasting in rats induced postoperative ER stress (the activation of IRE1A) and the repression of muscle GLUT4, leading to IR and hyperglycemia [69]. Finally, a recent study in the brain hippocampus of DM rats has associated the activation of ER stress (BIP, DDIT3 and ATF4) and inflammatory stress (tumor necrosis factor (TNF) and interleukin-6 (IL6)) with reduced GLUT4 expression; furthermore, metformin/donepezil treatment was demonstrated as efficacious to reverse these alterations, becoming a promising way to manage DM-associated dementia [70].

### 3.2. Atherogenesis/CVD and ER Stress/Inflammation

Hyperglycemia is known as a detrimental factor for the development of long-term complications of DM. In the association of CVD, DM, obesity and other metabolic diseases, altered lipid metabolism is an important etiopathogenic mechanism. The modification in lipid metabolism is both a biomarker, and a pathological factor, for CVD, T2D, obesity and other metabolic diseases; under these conditions, impaired cellular homeostasis hinders the proper functioning of the ER, and thus ER stress as well as inflammation play a fundamental role in the pathogenicity of these diseases (for a review, see [71]).

The scavenger receptor A, the platelet glycoprotein 4 (alias FAT and SCARB3), and the oxidized low-density lipoprotein receptor 1 are directly linked to ER stress, and their inhibition is a target to prevent the development of atherosclerosis [72,73,74]. Inflammatory and ER stress markers driven by the accumulation of free cholesterol and 7-ketocholesterol are increased in atherosclerotic lesion areas prone to rupture [30,75].

The chronic activation of ER stress is closely associated with endothelial dysfunction and atherosclerosis (for a review, see [76]). The accumulation of free cholesterol in cells, inflammation, and other associated CV risk factors (hyperhomocysteinemia, saturated fatty acids, modified lipoproteins and DM) trigger or worsen ER stress (for a review, see [77]). Conceivably, cholesterol is esterified by the sterol O-acyltransferase (alias ACAT), allowing the storage of esterified cholesterol in a relatively inert form in the cytosol and the rapid bioavailability of free cholesterol. In addition, it avoids the unspecific flux of free cholesterol among cell organelles that is especially toxic to the ER. The analysis of the transcriptome of aortic tissues in high-fat-diet-fed *Apoe*-knockout mice demonstrated the enhancement of three major UPR pathways, as well as 50 overlapping genes involved in UPR signaling, adaptation and apoptosis [78]. Furthermore, in DM *Apoe*-deficient mice, supplemented with glucosamine, larger and more advanced atherosclerotic lesions at the aortic root, displaying increased immunoreactivity for BIP and ENPL (endoplasmin, alias GRP94) at the lesion tissue, was observed [79]

Excess cholesterol and crystals of cholesterol also trigger the activation of the inflammasome, which together with the activation of ER stress are in an intricate connection that underlies the development and progression of atherosclerosis [80]. Interestingly, one of the mediators of UPR, the ER-resident ATF6, is similar to SREBP, activated by two proteolytic cleavages in the Golgi compartment [81]. It has been shown that ATF6, although inhibiting the activation of SREBP, is itself capable of inducing lipogenesis, establishing a link between UPR and dyslipidemia [82,83].

The activation of liver X receptor (LXR), by enhancing the expression of ABCA1 and ABCG1, improves the cholesterol efflux, alleviates ER stress and reduces apoptosis and defective efferocytosis in atherosclerotic lesion [84,85]. The pharmacological inhibition of cholesterol esterification and the stimulus to cholesterol efflux after treating with APOA1 mimetics, or overexpressing APOA1, abrogate ER and inflammatory stress [86,87]. Besides, by inhibiting oxidation and inflammation, and stimulating excess cholesterol efflux, HDL contributes to preventing ER stress [87]. Treatment of vascular smooth cells with a high-glucose medium induces the expression of CD36, cytokines and markers of calcification and ER stress; a condition that is aggravated by adding oxidized LDL. Those results were reinforced by findings in carotid plaques obtained from DM subjects in comparison to non-diabetic individuals [88].

## 4. AGEs-Induced and ER Stress/Inflammation-Mediated Effects in DM

Inflammation, oxidative stress and sterol accumulation, induced by AGE, trigger ER stress characterized by the enhanced expression of BIP, ENPL, EIF2A and ATF6A (cyclic AMP-dependent transcription factor ATF-6 alpha). Furthermore, glycation per se is a contributing factor to protein misfolding, eliciting UPR as an intracellular protein quality control system [89,90]. Glycation changes the electrostatic interactions and the hydrophobicity of polypeptide residues that trigger ubiquitination and degradation [91]. In fact, AGEs and ER stress often chronically coexist under many biological conditions, evincing their integrated action in the progression of DM- and other carbonyl stress-related complications. In addition, the propagation of this intricate mechanism is strengthened by noticing that the expression of AGER and its ligand—calgranulin S100 B—increases in neurological disorders, marked by increased ER stress activation [92].

The proteome of endothelial cells incubated with high-glucose medium identified the abundance of 331 proteins differentially expressed in comparison to the normoglycemic medium. Apart from the proteins directly involved in glycolysis and gluconeogenesis, the heat shock proteins related to UPR and protein refolding, the ubiquitin E2 ligases involved in protein degradation by the proteasomal and lysosomal system increased upon toxic glycoxidative stress mediated by MGO generation [93]. Recently, the plasma concentration of the tribbles homolog 3 protein (TRB3) (which is enhanced by hyperglycemia and ER stress) was found increased in subjects with T2D together with AGEs, BIP and TNF; moreover, it is positively correlated with fasting plasma glucose and AGEs [94].

### 4.1. AGEs and GLUT4 Expression Regulation

Advanced glycation has been extensively related to the development and progression of DM-induced degenerative complications; however, little is known about their participation in the glycemic homeostasis since, classically, they emerge only in situations of hyperglycemia. However, more recently, it has become evident that AGEs can be formed under other conditions of carbonyl stress, as well as obtained through exogenous sources, and that has led AGEs to occupy a key role in insulin sensitivity/GLUT4/glycemic homeostasis, closing a vicious circle. In fact, there is considerable evidence regarding the role of exogenous AGEs, mainly from dietary sources, in the induction of IR and DM in animal models and humans (for a review, see [95]).

In humans, long-term intervention trials with a high-AGE-containing diet are not used for ethical issues; nevertheless, studies with a low-AGE containing diet revealed amelioration of IR, which is, in part, ascribed to the enhanced expression of *DDOST* and *SIRTUIN 1*, due to their antioxidant and anti-inflammatory actions [96,97].

In vitro studies have contributed to demonstrating the direct effect of AGEs upon tissue insulin sensitivity, especially in GLUT4 regulation and inflammatory activity. AGER overexpression in 3T3-L1 preadipocytes accelerates adipocyte hypertrophy, whereas inhibition of AGER by small interfering RNA decreases adipocyte hypertrophy; furthermore, AGER-induced adipocyte hypertrophy was associated with the reduction of insulin signaling and glucose uptake, downregulation of GLUT4 and upregulation of toll-like receptor [98]. Additionally, in cultured human preadipocytes, adipogenesis was associated with increased levels of CML and AGER, and CML was seen to induce an AGER-dependent dysregulation of inflammatory adipokines [99]. Furthermore, a recent study confirmed that 3T3-L1 adipocytes cultured in a proglycating medium (with MGO or MGO-modified bovine serum albumin) increase *Ager* and reduces the *Slc2a4* gene expression; besides, bovine serum albumin-MGO reduces glucose uptake [100].

Interestingly, epicardial adipose tissue (EAT), a visceral fat surrounding the myocardium, which is potentially involved in the onset/progression of coronary artery disease, has also been investigated in subjects undergoing open-heart surgery. Increased AGER expression in EAT was observed to correlate with increased EAT thickness, reduced expression of GLUT4, adiponectin and lactoylglutathione lyase (alias glyoxalase 1), and increased expression of the high-mobility group protein B1, toll-like receptor and MYD88 (myeloid differentiation primary response protein MyD88). These data indicate that, in subjects with coronary disease, AGER may be involved in promoting EAT adiposity and metabolic dysfunction, and that is related to the decreased GLUT4 expression [101].

The first indication of a primary in vivo effect of AGE upon glycemic homeostasis and GLUT4 expression was reported in experimental healthy Sprague-Dawley rats subjected to chronic administration of MGO. In this model, increased plasma glucose, reduced GLUT4 expression and glucose uptake (adipose tissue) and severe beta cell damage were observed, evincing a classic T2D profile [102]. Later, in healthy Wistar rats, chronic administration of AGE-albumin (3 months) was reported to increase adiposity and body weight as well as to induce whole-body IR, associated with an increased expression of inflammatory markers and decreased expression of *Scl2a4*/Glut4 in periepididymal adipose tissue [103].

Finally, we have investigated the participation of ER stress and inflammation in the in vivo and in vitro effects of AGEs in the *Slc2a4*/GLUT4 expression in skeletal soleus muscle of healthy rats [9]. For in vivo analysis, rats were treated (12 weeks) with AGE-albumin, and developed whole-body IR, with decreased *Slc2a4*/GLUT4 expression, increased nuclear content of NFKB (p50) and increased cellular content of BIP [8]. For in vitro analysis, muscles from healthy rats were incubated (2.5 to 7.5 h) with AGE-albumin, and displayed a decreased *Slc2a4*/GLUT4 content, increased BIP and ENPL contents, as well as increased phosphorylation of IKKA and IKKB and nuclear NFKB (p50 and p65) content; furthermore, EMSA analysis revealed an increase in nuclear protein binding in the NFKB-binding site of the *Slc2a4* promoter [9].

**Figure 1:**
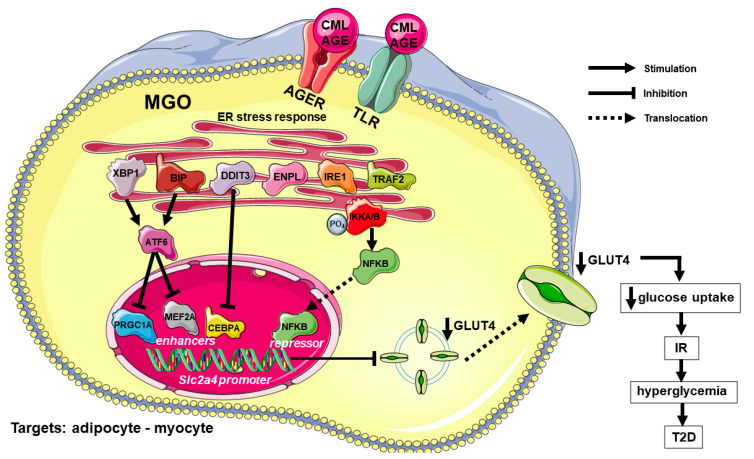
Advanced glycation end products (AGE)-induced regulation of glycemic homeostasis and glycemic homeostasis: participation of inflammatory and endoplasmic reticulum stress. In adipose and muscle tissues, AGE/CML binding to AGER and TLR activate the ER-stress response, including increased expression and/or activation of BIP, ENPL, XBP1, DDIT3, IRE1 and TRAF2. XBP1 and BIP activate ATF6, which is a repressor of the transcription factor PRGC1A and MEF2A (enhancers of Slc2a4 gene), thus decreasing Slc2a4 expression. On the other, IRE1/TRAF2 activation overlap the inflammatory pathway phosphorylating IKKA/B, and promoting NFKB dissociation and migration into the nucleus, where it plays a potent repressor effect upon Slc2a4 gene expression. Besides, intracellular MGO can contribute to the increase in the activity of proinflammatory cytokines, reinforcing this pathway. These effects on the Slc2a4 gene expression decreases GLUT4 synthesis and plasma membrane content, eventually decreasing insulin-induced glucose uptake, and establishing IR. In turn, IR leads to hyperglycemia, which can cause T2D or worsen preexisting DM. Symbols are: AGE, advanced glycated end product; AGER, advanced glycation end product-specific receptor, alias RAGE; ATF6, cyclic AMP-dependent transcription factor ATF; BIP, endoplasmic reticulum chaperone BIP, alias GRP78; CEBPA, CCAAT enhancer binding protein alpha; CML, carboxymethyl-lysine; DM, diabetes mellitus; DDIT3, DNA damage-inducible transcript 3 protein, alias CHOP10/GADD153; DM, diabetes mellitus; ENPL, endoplasmin, alias GRP94; ER, endoplasmic reticulum; GLUT4, solute carrier family 2, facilitated glucose transporter member 4; IKKA/B, inhibitor of nuclear factor kappa-B kinase subunits alpha/beta; IR, insulin resistance; IRE1, inositol requiring enzyme-1; MGO, methylglyoxal; MEF2A, myocyte enhancer factor 2A; NFKB, nuclear factor NF-kappa-B; PRGC1A, peroxisome proliferator-activated receptor gamma coactivator 1-alpha, alias PGC1A; Slc2a4, solute carrier family 2 member 4 gene; T2D, type 2 diabetes mellitus; TLR, toll-like receptors; TRAF2, TNF receptor-associated factor 2; XBP1, X-box-binding protein 1. Names and symbols of proteins are in accordance with the UNIPROT database (https://www.uniprot.org/, accessed on 2 November 2021). Parts of the figure were drawn from Servier Medical Art (https://smart.servier.com/, accessed on 2 November 2021).

These studies reveal that high AGE concentrations impair glucose homeostasis through a mechanism that involves ER stress/inflammation-mediated repression of the *Slc2a4*/GLUT4 expression. Figure 1 summarizes the main mechanisms involved in the AGE-induced and ER stress-mediated repression of GLUT4 expression and the impairment of glycemic homeostasis.

### 4.2. AGEs and Atherogenesis/CVD Development

Inflammation has an important role in the development of CVD, which can be initially observed by its role in the macrophage homeostasis. Albumin, the most abundant protein in circulation, has enhanced susceptibility to glycation, and AGE-albumin increases the generation of ROS in macrophages, priming these cells to the lipopolysaccharide-induced secretion of inflammatory cytokines [104], and inducing the accumulation of oxysterols [105].

In non-diabetic dyslipidemic mice (*Apoe*-knockout mouse), treatment with AGE-albumin increased lipid deposition in the aortic arch, enhanced the expression of *Ager*, *Tnf* (*tumor necrosis factor*) and *Nox4* (*NADPH oxidase 4*) genes, and increased the marker of lipid peroxidation (4-hydroxynonenal) and CML, in comparison with animals receiving control-albumin. Interestingly, treatment with losartan (antagonist of the angiotensin 2 receptor) abrogated lipid accumulation and expression of the AGE–AGER axis in the aortic arch of animals treated with AGE-albumin [106].

The participation of ER stress in the development of CVD has been firstly characterized by its role in macrophage homeostasis, a field we have contributed to in the last decade. Oxidative stress induces a reduction in the ABCA1 protein level and cholesterol efflux to APOA1; both events are avoided by incubating cells with aminoguanidine, an antiglycation and antioxidation compound that reduces ROS generation [107]. Besides, the use of a chemical chaperone (4-phenylbutyric acid), which alleviates ER protein misfolding, recovers the ABCA-1 levels [108]. The ER-associated degradation (ERAD) pathway is involved in the degradation of ABCA1, together with the endosomal sorting complex required for transport pathway and the proteolysis mediated by surface calpains. [109].

Macrophages treated with AGE-albumin (produced in vitro and isolated from subjects with poorly controlled DM) have a faster intracellular degradation of ABCA1, due to the activity of ubiquitin-proteasomal and lysosomal systems, which are independent of surface calpains [110]. The effects on disturbing the APOA1-mediated cholesterol removal and inducing inflammation persisted even after a long period of cell resting in the absence of AGE-albumin. In addition, the impairment of cholesterol efflux was restored after the improvement of the glycemic control, reflected by diminished levels of AGEs [111]. In the absence of AGER in macrophages, attained by AGER silencing or using peritoneal macrophages from *Ager* knockout mice, the deleterious effects of AGE-albumin produced in vitro or isolated from subjects with DM were abrogated. Moreover, the altered expression of genes involved in lipid efflux was restored in the absence of AGE/AGER signaling [112]. Considering the ability of many other receptors to interact with AGEs, further studies are necessary to better understand the blockage of AGE signaling in the improvement of cholesterol balance in the arterial wall.

Diabetic kidney disease is a prevalent complication of DM that increases cardiovascular mortality. AGEs are also prevalent in chronic kidney disease due to the failure in the detoxification of the Maillard reaction intermediates. Besides, carbamoylation occurs because of urea dissociation into thiocyanate that reacts with proteins. In an animal model of uremia, carbamoylated albumin disturbed cholesterol efflux, which was associated with ER stress [113]. In humans with diabetic kidney disease, albumin carbamoylation enhances according to the reduction in estimated glomerular filtration rate and diminishes HDL-mediated cholesterol efflux from macrophages [114]. Regarding the potential mechanisms involved, it has been reported that AGE-albumin increases the expression of BIP and induces apoptosis dependent of the intracellular calcium increase in kidney podocytes; furthermore, TUDCA, which impairs ER stress by acting as a chaperone, prevents the cell from dying [115]. Furthermore, MGO-derived AGEs were confirmed to induce ER stress (increased BIP, ATF4 and DDIT3) in kidney cells, associated with inflammation, apoptosis and an imbalance in mitochondrial function [116].

**Figure 2:**
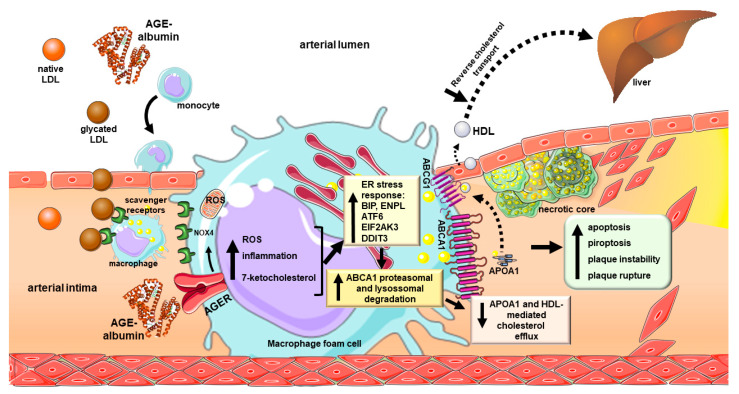
Advanced glycation end products-induced atherogenesis and CVD development: participation of inflammatory and endoplasmic reticulum stress. Glycated LDL reaches the arterial intima inducing circulating monocyte chemotaxis. Differentiated macrophages take up modified LDL by the scavenger receptors which induces intracellular cholesterol accumulation. In addition, advanced glycated albumin (AGE-albumin), modified in blood circulation or in the arterial intima is taken up by AGER. This induces the generation of ROS by NOX4 and mitochondrial respiratory chain activation. Moreover, AGEs induce inflammation and ER stress, with increased expression of BIP, ENPL, ATF6, EIF2AK3 and DDIT3 that relates to ABCA1 degradation by the ubiquitin-proteasomal and lysosomal systems. Ultimately, a dramatic decrease in ABCA1 protein content in macrophage foam cells is observed, leading to the reduction in the APOA1 and pre-beta HDL-mediated cholesterol efflux. Although AGEs do not alter ABCA1 mRNA, they reduce the gene expression of ABCG1, by an LXR-dependent mechanism, then compromising the HDL-mediated cholesterol efflux. The accumulation of cholesterol and oxysterols perpetuates the generation of ROS, inflammatory cytokines and AGEs, which culminates in a necrotic core formation, with apoptosis, pyroptosis, plaque instability and rupture. The deleterious effects of AGEs on macrophage homeostasis damage the cholesterol flux to the liver by the reverse cholesterol transport contributing to the independent relation of AGEs with CVD. Symbols are: ABCA1, phospholipid-transporting ATPase ABCA1; ABCG1, ATP binding cassette transporter G-1 AGE, advanced end product; AGER, advanced glycation end product-specific receptor, alias RAGE; APOA1, apolipoprotein A-1; ATF6, cyclic AMP-dependent transcription factor ATF-6; BIP, endoplasmic reticulum chaperone BIP, alias GRP78; CVD, cardiovascular disease; DDIT3, DNA damage-inducible transcript 3 protein, alias CHOP10/GADD153; EIF2AK3, eukaryotic translation initiation factor 2-alpha kinase 3, alias PERK; ENPL, endoplasmin, alias GRP94; HDL, high-density lipoprotein; LDL, low-density lipoprotein; LXR, liver X receptor; NOX4, NADPH oxidase 4; ROS, reactive oxygen species. Names and symbols of proteins are in accordance with the UNIPROT database (https://www.uniprot.org/, accessed on 2 November 2021). Parts of the figure were drawn from Servier Medical Art (https://smart.servier.com/, accessed on 2 November 2021).

In animal model of DM, the activation of EIF2AK3 was observed as involved in the AGE-induced coronary dysfunction [117]; furthermore, AGE-induced activation of ER stress was also related to post-myocardial infarction ventricular arrhythmias [118]. Glycated LDL induces intense ER stress in bovine aortic endothelial cells, activating BIP, EIF2AK3 and ATF6, which was accompanied by impaired endothelium-dependent vasodilation [119]. Moreover, in human vascular endothelial cells, the reduction in the antioxidant enzyme paraoxonase 2 elicited by AGEs is associated with increased oxidative, inflammatory and ER stress [120]. Finally, a beneficial effect of lowering the AGEs levels for CVD in humans was suggested in the recent CORDIOPREV study, in which the reduction in circulating AGEs achieved by the Mediterranean diet, expressed by the MGO levels, was associated with a greater probability of T2D remission in subjects with CVD [121]. Figure 2 summarizes the main mechanisms involved in the AGE-induced and ER stress-mediated atherogenesis and CVD development.

## 5. AGEs, ER Stress and Inflammation in Non-Alcoholic Fatty Liver Disease (NAFLD)

As the knowledge of ER stress and inflammation advances, their involvement in several chronic diseases, such as DM, CVD, neurodegenerative disorders, fatty liver disease, inflammatory bowel and cancer, among others, become increasingly evident (for a review, see [122]). NAFLD has received considerable attention as a complication of DM, because of its increasing incidence and severity, and ER and inflammatory stress have been demonstrated to be involved in this condition.

Diabetes mellitus *Apoe*-knockout mice, supplemented with glucosamine, developed intracellular lipid accumulation, indicating the development of NAFLD, and that was associated with increased content of the UPR markers BIP, ENPL, DDIT3 and protein disulfide-isomerase in liver tissue [79]. On the other hand, we have reported that T2D mice develop histological signals of non-alcoholic steatohepatitis (NASH, according to NASH-CRN Pathology Committee System), associated with impaired glucose metabolism in the liver, and increased expression of the inflammatory markers TNF, IL6 and NFKB, as well as increased binding activity of nuclear proteins in NFKB-binding site target genes, as measured in the *Slc2a2* (solute carrier family 2 member 2) gene [123].

The P2Y purinergic receptor 12, an adenosine diphosphate responsive G protein-coupled receptor expressed on the surface of platelets (required for normal platelets aggregation), is also expressed by macrophages in the liver from mice with cirrhosis and hepatocellular carcinoma. Ticagrelor-induced inhibition of P2Y purinergic receptor 12 enhances tumor cell phagocytosis by macrophages and induces an anti-tumoral phenotype, which was associated with the increased expression of several actors of the ER stress pathways, indicating activation of the UPR [124]. Besides, the inhibition of the UPR with TUDCA diminishes the pro-phagocytotic effect of ticagrelor, confirming that P2Y purinergic receptor 12 mediates macrophage function through the activation of ER stress [124]. This seems to be relevant in the pathogenesis of chronic liver disease and cancer.

Increased consumption of sugars, especially fructose, and AGE-enriched diets are related to the generation of intracellular AGEs that induce lipogenesis and inflammation in crosstalk with ER stress [125]. Besides, the extravasation of AGEs and its interaction with RAGE is associated with the development and evolution of several chronic diseases, including steatosis and CVD [126]. A recent meta-analysis found an association among elevated levels of several types of AGEs with NAFLD [127] and the determination of fluorescent AGE has been considered as a potential biomarker for NAFLD stratification [128].

In sum, nowadays, the interplay among AGEs, ER stress and inflammation in the development and/or progression of NAFLD in DM becomes increasingly more evident (for a review, see [129,130]).

## 6. Concluding Remarks

Over the last two decades, extensive knowledge of the molecular players in the pathways of ER and inflammatory stress has been gained; at the same time, their participation in the etiopathogenesis and pathophysiology of innumerous diseases has also grown. Interestingly, the sharing of some players through the two pathways has been increasingly demonstrated, evincing a very complicate and intricate interplay between ER and inflammatory stress.

Endoplasmic reticulum and inflammatory stress are recurrently found to be involved in the pathophysiology of DM, whether in mechanisms related to glycemic control (insulin secretion and action) or in the development/progression of DM complications. Furthermore, hyperglycemia increases the generation of AGEs, which in turn refeed ER and inflammatory stress, contributing to the impairment of glycemic homeostasis and the acceleration of DM complications.

In this article, we have reviewed the molecular mechanisms involved in the AGEs-induced activation of ER and inflammatory stress pathways, leading to the repression of the *Slc2a4* gene and consequently to the decrease in GLUT4 content in adipose and muscle tissue. This event reduces plasma glucose clearance and contributes to hyperglycemia, establishing a dangerous vicious circle in DM. Additionally, AGEs also induce, by an ER stress/inflammation-mediated way, the development of atherogenesis, contributing to the progression of CVD.

To date, in DM, it is clear that the impairment of glycemic homeostasis and the development of degenerative complications have important AGEs-induced and ER stress/inflammation-mediated components. Experimentally, the blockage of some ER and inflammatory stress players in specific tissues has exhibited excellent results for the improvement of glycemic control or the inhibition of CVD development/progression. Thus, based on ER and inflammatory stress inhibition, several potential targets for the prevention and/or treatment of DM and its complications can be developed in the future.

## List of Abbreviations and Acronyms

Names and symbols of genes and proteins referred to in this manuscript are in accordance with the HGNC (https://www.genenames.org/, accessed on 2 November 2021) and the UNIPROT (https://www.uniprot.org/, accessed on 2 November 2021) databases.

ABCA1	the phospholipid-transporting ATPase ABCA1
ABCG1	ATP binding cassette transporter G-1
AGE	advanced glycation end products
AGER	advanced glycation end product-specific receptor, (alias RAGE)
APOA1	apolipoprotein A-1
APOB	apolipoprotein B
*Apoe*	*apolipoprotein E gene* (rat/mouse)
ATF4	cyclic AMP-dependent transcription factor ATF-4
ATF6	cyclic AMP-dependent transcription factor ATF-6
*Atf6*	*activating transcription factor 6 gene* (rat/mouse)
ATF6A	cyclic AMP-dependent transcription factor ATF-6 alpha
BIP	endoplasmic reticulum chaperone BIP (alias GRP78)
CD36	platelet glycoprotein 4 (alias FAT/SCARB3)
CEBPA	CCAAT enhancer binding protein alpha
*Cebpa*	*CCAAT enhancer binding protein alpha* gene (rat/mouse)
CML	carboxymethyllysine
CVD	cardiovascular disease
DDIT3	DNA damage-inducible transcript 3 protein (alias CHOP10/GADD153)
*Ddit3*	*DNA damage-inducible transcript 3* gene (rat/mouse)
DDOST	dolichyl-diphosphooligosaccharide-protein glycosyltransferase, alias AGER1
*DDOST*	*dolichyl-diphosphooligosaccharide-protein glycosyltransferase* gene (human)
DM	diabetes mellitus
EAT	epicardial adipose tissue
EIF2A	eukaryotic translation initiation factor 2A
EIF2AK3	eukaryotic translation initiation factor 2-alpha kinase 3 (alias PERK)
EMSA	electrophoretic mobility shift assay
ENPL	endoplasmin (alias GRP94)
ER	endoplasmic reticulum
GLUT4	solute carrier family 2, facilitated glucose transporter member 4
HDL	high-density lipoprotein
HSP	heat shock protein
HSP70	heat shock 70 kDa protein
*Hspa5*	*heat shock protein family A member 5* gene (rat-mouse)
IKKA	inhibitor of nuclear factor kappa-B kinase subunits alpha
IKKB	inhibitor of nuclear factor kappa-B kinase subunits beta
IL6	interleukin-6
IR	insulin resistance
IRE1	inositol requiring enzyme 1
LDL	low-density lipoprotein
LXR	liver X receptor
MEF2A	myocyte enhancer factor 2A
MGO	methylglyoxal
MYD88	myeloid differentiation primary response protein MyD88
NAFLD	non-alcoholic fatty liver disease
NASH	non-alcoholic steatohepatitis
NFKB	NFKB nuclear factor-kappa-B
*Nfkb*	*nuclear factor-kappa-B gene* (rat/mouse)
*Nox4*	*NADPH oxidase 4 gene* (rat/mouse)
PM	plasma membrane
PRGC1A	peroxisome proliferator-activated receptor gamma coactivator 1-alpha (alias PGC1A)
ROS	reactive oxygen species
*Slc2a2*	*solute carrier family 2 member 2 gene* (rat/mouse)
*Slc2a4*	*solute carrier family 2 member 4 gene* (rat/mouse)
*SLC2A4*	*solute carrier family 2 member 4 gene* (human)
SREBP1	sterol regulatory element-binding protein
T1D	type 1 diabetes mellitus
T2D	type 2 diabetes mellitus
TRAF2	TNF receptor-associated factor 2
TRB3	tribbles homolog 3 protein
TNF	tumor necrosis factor
*Tnf*	*tumor necrosis factor* gene (rat/mouse)
TUDCA	tauroursodeoxycholic acid
UPR	unfolded protein response
VLDL	very low-density lipoprotein
